# F119. MULTILEVEL ANALYSIS IMPROVES THE MODEL FIT OF THE DIMENSIONAL STRUCTURE OF THE PANSS IN PATIENTS WITH SCHIZOPHRENIA

**DOI:** 10.1093/schbul/sby017.650

**Published:** 2018-04-01

**Authors:** Cinthia Higuchi, Hugo Cogo-Moreira, Bruno Bertolucci, Christoph U Correll, Cristiano Noto, Quirino Cordeiro, Rosana Freitas, Hélio Elkis, Sintia I Belangero, Rodrigo A Bressan, Ary Gadelha

**Affiliations:** 1 Universidade Federal de São Paulo; 2 The Zucker Hillside Hospital, Hofstra North Shore LIJ School of Medicine; 3 Faculdade de Ciências Médicas da Santa Casa de São Paulo; 4 Universidade de Sao Paolo

## Abstract

**Background:**

Principal component analyses (PCA) studies show that schizophrenia symptoms are usually grouped into five domains. However, to infer a latent dimensional structure, confirmatory factor analysis (CFA) is more appropriate than PCA. Most CFA studies addressing the five-factor model yielded poor fit indices. One single study achieved a good fit using a multilevel CFA structure with the interviewers as level. Other possible reasons for sample heterogeneity and subsequent poor model adjustments, such as differences in patients’ clinical profiles across clinical units and clinical staging, were not measured in this study. We aimed to replicate the effect of the CFA multilevel analyses and evaluate the possible influence of other heterogeneity sources as levels, i.e., clinical staging, on the Positive and Negative Syndrome Scale (PANSS) five-factor structure.

**Methods:**

700 patients with schizophrenia at four different centers had their PANSS analyzed. A Confirmatory Factor Analysis (CFA) was conducted using the following fit index: Comparative Fit Index (CFI) and Non-Normed Fit Index (NNFI) >0.95, the Root Mean Square Errors of Approximation (RMSEA) <0.06, and Weighted Root Mean Square Residual (WRMR) <1.0. Thereafter, we performed multilevel analyses considering the following levels: i) centers, ii) interviewers and iii) clinical staging for schizophrenia (first episode, treatment-resistant schizophrenia and non-treatment resistant schizophrenia).

**Results:**

The mean (SD) age was 34.9 (10.3) years, mean age of onset was 21.7 (7.5), mean duration of illness means was 13.2 (9.7) years, and 64.3% of the sample was male. The CFA model without multilevel analyses yielded poor fit indices: RMSEA = 0.102 (90% CI: 0.097 – 0.107; Cfit was <0.001), CFI = 0.921 and NNFI = 0.906 and WRMR = 1.952. When the multilevel analysis was applied, all models reached an acceptable fit: i) centers: RMSEA = 0.044 (90% CI: 0.038 – 0.049; CFit = 0.964), CFI = 0.981, NNFI = 0.977, and WRMR = 1.860; ii) interviewers: RMSEA = 0.047 (90% CI: 0.041 – 0.053; CFit = 0.765), CFI = 0.947, NNFI = 0.938, and WRMR = 1.531; iii) clinical stage: RMSEA = 0.052 (90% CI: 0.046 – 0.058; CFit = 0.274), CFI = 0.988, NNFI = 0.985, and WRMR = 2.433.

**Discussion:**

Good CFA model fits were only achieved when the multilevel structure was applied. Besides the bias generated by data collection (i.e., local of data collection and raters), the clinical staging is a potential source of variability to consider in schizophrenia dimensional structure. As dimensional approaches gain relevance to reduce heterogeneity in schizophrenia and to investigate their biological substrates, reliable methods to address latent dimensions are required.

